# Membrane Potential and Calcium Dynamics in Beta Cells from Mouse Pancreas Tissue Slices: Theory, Experimentation, and Analysis

**DOI:** 10.3390/s151127393

**Published:** 2015-10-28

**Authors:** Jurij Dolenšek, Denis Špelič, Maša Skelin Klemen, Borut Žalik, Marko Gosak, Marjan Slak Rupnik, Andraž Stožer

**Affiliations:** 1 Institute of Physiology, Faculty of Medicine, University of Maribor, SI-2000 Maribor, Slovenia; E-Mails: jurij.dolensek@um.si (J.D.); masa.skelin@um.si (M.S.K.); marko.gosak@um.si (M.G.); marjan.rupnik@um.si (M.S.R.); 2 Faculty of Electrical Engineering and Computer Science, University of Maribor, SI-2000 Maribor, Slovenia; E-Mails: denis.spelic@um.si (D.Š.); borut.zalik@um.si (B.Ž.); 3 Center for Open Innovation and Research, Core@UM, University of Maribor, SI-2000 Maribor, Slovenia; 4 Department of Physics, Faculty of Natural Sciences and Mathematics, University of Maribor, SI-2000 Maribor, Slovenia; 5 Center for Physiology and Pharmacology, Medical University of Vienna, A-1090 Vienna, Austria

**Keywords:** calcium sensors, membrane potential sensors, calcium imaging, membrane potential imaging, beta cell, pancreas, denoising, patch-clamp

## Abstract

Beta cells in the pancreatic islets of Langerhans are precise biological sensors for glucose and play a central role in balancing the organism between catabolic and anabolic needs. A hallmark of the beta cell response to glucose are oscillatory changes of membrane potential that are tightly coupled with oscillatory changes in intracellular calcium concentration which, in turn, elicit oscillations of insulin secretion. Both membrane potential and calcium changes spread from one beta cell to the other in a wave-like manner. In order to assess the properties of the abovementioned responses to physiological and pathological stimuli, the main challenge remains how to effectively measure membrane potential and calcium changes at the same time with high spatial and temporal resolution, and also in as many cells as possible. To date, the most wide-spread approach has employed the electrophysiological patch-clamp method to monitor membrane potential changes. Inherently, this technique has many advantages, such as a direct contact with the cell and a high temporal resolution. However, it allows one to assess information from a single cell only. In some instances, this technique has been used in conjunction with CCD camera-based imaging, offering the opportunity to simultaneously monitor membrane potential and calcium changes, but not in the same cells and not with a reliable cellular or subcellular spatial resolution. Recently, a novel family of highly-sensitive membrane potential reporter dyes in combination with high temporal and spatial confocal calcium imaging allows for simultaneously detecting membrane potential and calcium changes in many cells at a time. Since the signals yielded from both types of reporter dyes are inherently noisy, we have developed complex methods of data denoising that permit for visualization and pixel-wise analysis of signals. Combining the experimental approach of high-resolution imaging with the advanced analysis of noisy data enables novel physiological insights and reassessment of current concepts in unprecedented detail.

## 1. Introduction: Beta Cell as a Sensor

Beta cells from the pancreatic islets of Langerhans are a crucial functional element in the negative feedback loop controlling plasma concentration of energy-rich nutrients, such as glucose, amino acids, and fatty acids. These cells serve as biological sensors which detect increases in the concentration of fuel molecules, and through a series of events collectively termed “stimulus-secretion coupling” they respond by secreting insulin [1,2,3,4,5]. Insulin, as an anabolic hormone, acts on cells within target organs, such as the liver, skeletal muscle, and adipose tissue, promoting use, uptake, and storage of energy-rich nutrients and effectively completing the feedback loop [6,7]. A substantial lack of insulin effects manifests itself clinically as diabetes mellitus and studying and understanding the stimulus-secretion coupling in ever-increasing detail is crucial to understanding the pathophysiology of diabetes mellitus and to find new treatment modalities [8,9]. Here, we briefly summarize the crucial steps in beta cell stimulus-secretion coupling to provide a logical framework for the following chapters on experimental and analytical approaches to studying this process.

The fuel secretagogues glucose, amino acids, and fatty acids enter the beta cell via glucose transporters, amino acid transporters, and by diffusion, respectively, and are subsequently metabolized in the cytosol and mitochondria. The metabolism of fuels seems to be the necessary condition for fuel-induced insulin secretion (FIIS) and yields a number of different intermediates and cofactors that mediate the stimulus-secretion coupling process and are collectively termed metabolic coupling factors (MCFs) [10,11,12]. Glucose is the principal fuel secretagogue and induces the so-called glucose stimulated insulin secretion (GSIS) also termed glucose induced insulin secretion (GIIS). GIIS consists of two principal pathways: a triggering and an amplifying pathway. The first transduces an increase in concentration of glucose to an increase in intracellular calcium ion (Ca^2+^) concentration ([Ca^2+^]_i_), which triggers insulin secretion [1,2,5,12]. It involves metabolism of glucose via glycolysis and mitochondrial oxidation yielding adenosine triphosphate (ATP), an ATP-induced decrease in open probability of ATP-dependent potassium (K_ATP_) channels, the subsequent plasma membrane depolarization, and opening of voltage-dependent calcium channels (VDCCs). The influx of Ca^2+^ then produces an increase in intracellular calcium concentration, thereby triggering exocytosis of insulin-containing granules [1,2,4,5,13]. The triggering signal can be further modulated by uptake and release of Ca^2+^ into and from a number of intracellular stores and the dynamics of global and local [Ca^2+^]_i_ changes may differ [2,14,15,16]. ATP is an example of what is called an effectory MCF since its target (the K_ATP_ channel) is a membrane effector protein [12]. The amplifying pathway on the other hand requires the triggering Ca^2+^ signal but promotes insulin secretion distally of changes in [Ca^2+^]_i_ [1,2,4,16]. The effectory MCFs in this pathway are less well established, but ATP, reduced nicotinamide adenine dinucleotide phosphate (NADPH), and cyclic adenosine monophosphate (cAMP) are some of the candidates [1,12] and their putative modes of action involve increasing the release competence of secretory granules and the likelihood of their undergoing exocytosis in response to a given Ca^2+^ signal [4,14]. In addition to the effectory MCFs of the triggering and amplifying pathway which act distally in the stimulus-secretion coupling process by directly influencing effector proteins, the so called regulatory MCFs act upstream in the stimulus-secretion coupling process and influence key intracellular metabolic processes. For instance, in GIIS citrate is an important regulatory MCF and influences Krebs cycle activity, but also the activity of two additional metabolic cycles that generate MCFs, the pyruvate cycle and the glycerolipid/free fatty acid cycle [11,12].

The other two classes of fuels also generate MCFs that play a central role in the *in vivo* setting where mixed meals, rather than glucose alone, are sensed by the beta cell. Fatty acids are not sufficient to provide the triggering stimulus and this is especially important in the fasted state when fatty acids are metabolized via beta oxidation and intracellular lipid MCFs do not accumulate [10,11]. Postprandially, glucose inhibits beta oxidation (via malonyl-coenzyme A), provides glycerol triphosphate for esterification, and activates lipolysis, which together with free fatty acids provide MCFs for insulin secretion [10,11]. Amino acids are able to induce insulin secretion, especially in certain combinations, and they also importantly augment GIIS. Alanine and arginine are able to depolarize the beta cell upon entry and likely contribute to the triggering pathway. The metabolism of alanine and other amino acids also yields MCFs that support GIIS [11]. Finally, the metabolic pathways of glucose, FFAs, and AAs are strongly interconnected and details on MCFs, the metabolic cycles, as well as their interplay are covered in detail in exhaustive reviews [10,11,12,17,18,19,20,21,22].

To complicate things further, fuel secretagogues may influence intracellular signaling pathways via membrane receptors. Glucose can stimulate metabolism in the beta cell via the sweet taste receptor T1R3 [23], and fructose can promote insulin secretion via the T1R2 receptor [24], reviving the decade-old idea that the effects of glucose upon the beta cell are mediated via membrane receptors [25] and defining the so called sweet taste receptor pathway in beta cell stimulus-secretion coupling [26]. Moreover, the FFA receptor GPR40/FFAR1 is probably responsible for approximately half of the FFA-induced insulin secretion [27,28,29,30] and the heterodimeric amino acid taste receptor Tas1R1/Tas1R3 may be responsible for a part of glutamate- and arginine-induced insulin secretion [31].

Beta cells receive paracrine input from other islet cell types [32,33,34,35] and islets are richly perfused and innervated [36,37,38,39,40,41,42], therefore *in vivo* GIIS is modulated by hormones, such as somatostatin, glucagon, glucose-dependent insulinotropic peptide (GIP) and glucagon-like-peptide-1 (GLP-1), as well as by neurotransmitters, such as acetylcholine, noradrenaline, glutamate, and gamma-amino butyric acid (GABA). Somatostatin inhibits cAMP production via G_i/o_ protein-coupled SSTR2 and SSTR5 somatostatin receptors [43], whereas glucagon, GIP, and GLP-1 raise the concentration of intracellular cAMP via membrane G_s_ protein-coupled receptors [44,45]. Acetylcholine increases [Ca^2+^]_i_ through the muscarinic M3 and M5 receptors [46,47], noradrenaline predominantly inhibits insulin secretion by inhibiting cAMP production via G_i/o_ protein-coupled α-2 adrenergic receptors [45,48], glutamate possibly limits the duration of MP and [Ca^2+^]_i_ oscillations via the NMDA receptor [49,50], and GABA may stimulate insulin secretion by membrane depolarization via the ionotropic GABA_A_ receptor which functions as a chloride channel [51,52] or inhibit insulin secretion via the metabotropic GABA_B_ receptor which is coupled with the G_i/o_ protein [52,53]. Together, these influences constitute the so-called neurohormonal pathway [15,26].

Finally, in addition to fuel and endogenous neurohormonal secretagogues, pharmacological substances can be employed to influence beta cell stimulus-secretion coupling. So far, the only two approved classes of small molecules that directly target the beta cell are sulphonylureas and glinides, which induce insulin secretion via inhibition of the KATP channel independently of glucose, producing the triggering signal [45,54,55]. Additionally, sulphonylureas also influence the amplification pathway [54,56,57,58]. Due to their glucose-independence, both sulphonylureas and glinides are associated with the risk of hypoglycemia [59].

So far, our discussion has assumed a paradigmatic or average beta cell including all of the abovementioned pathways. In an islet of Langerhans, approximately a thousand heterogeneous beta cells are coupled through gap junctions, which reduces the heterogeneity and improves their functional responses [60,61,62,63,64,65,66,67,68,69], but the coupled cells retain some heterogeneity which allows for at least partly selective and gradual regulation of their function. Consequently, there is no such thing as an average beta cell and a complete picture of the physiological function of islets can only be understood by assessing the information flow throughout interconnected beta cells that leads to coordinated activity of cell populations and regulated hormone release [60,65,68,70,71,72,73]. With the advance in experimental techniques and computational abilities, studies that regard ensembles of beta cells as networks of interconnected dynamical elements are therefore gaining prominence [70,72,73,74,75,76,77]. Moreover, recently we and others studied populations of beta cells by means of graph-theoretical approaches and, thereby, succeeded in showing that the beta cells form a complex network [78]. The extracted non-trivial topological features importantly determine the heterogeneity of individual cells [79,80], and can be modulated by physiological [48,81] and pathophysiological influences [82,83,84].

In order to reliably dissect the differential effects of various secretagogues upon different crucial functional parameters in a given beta cell, such as MCFs, MP, [Ca^2+^]_i_, and exocytosis, we need methods that enable simultaneous measurements of all the given parameters or at least two of the parameters at a time. Since the dynamics of these parameters may be fast, the method should have a sufficient temporal resolution. Moreover, if we want to assess whether each of the above pathways is equally important in every beta cell in an islet of Langerhans, we need to study many beta cells at a time. In the following chapters, we will briefly present the tissue slice method and its combination with the classical electrophysiological patch-clamp technique [85] and CCD-camera-based imaging [86], as well as the modern confocal imaging modalities [86,87] to detect changes in MP and [Ca^2+^]_i_.

Recently, methods have become available to measure the most distal event in beta cell stimulus-secretion coupling, *i.e.*, exocytosis in many beta cells at a time [88,89,90,91,92,93,94,95]. Analogously, methods exist that enable detection of some of the crucial MCFs, e.g., ATP, cAMP, and NADPH, involved in more proximal metabolic steps in stimulus-secretion coupling [96,97,98]. However, they have not yet been combined with each other or with methods to detect MP or [Ca^2+^]_i_ with sufficient temporal and spatial resolution in tissue slices and, thus, remain beyond the scope of this article. Successfully studying proximal, intermediate, and more distal steps in the transduction pathway simultaneously in a large number of cells will shed light on the importance of various events in the stimulus secretion coupling during different phases of insulin secretion, in different cells, and for different physiological and pharmacological secretagogues. Due to possible differences in rodent and human beta cell structure and function [38,99,100,101,102,103,104,105,106], findings obtained in mice will have to be validated on human tissue slices [107,108].

## 2. Part I: Assessing Beta Cell Function Using MP and [Ca^2+^]_i_ Sensors

### 2.1. Measuring MP with a Patch-Clamp Pipette and [Ca^2+^]_i_ with Fluorescent Dyes Using a CCD Camera

**Figure 1:**
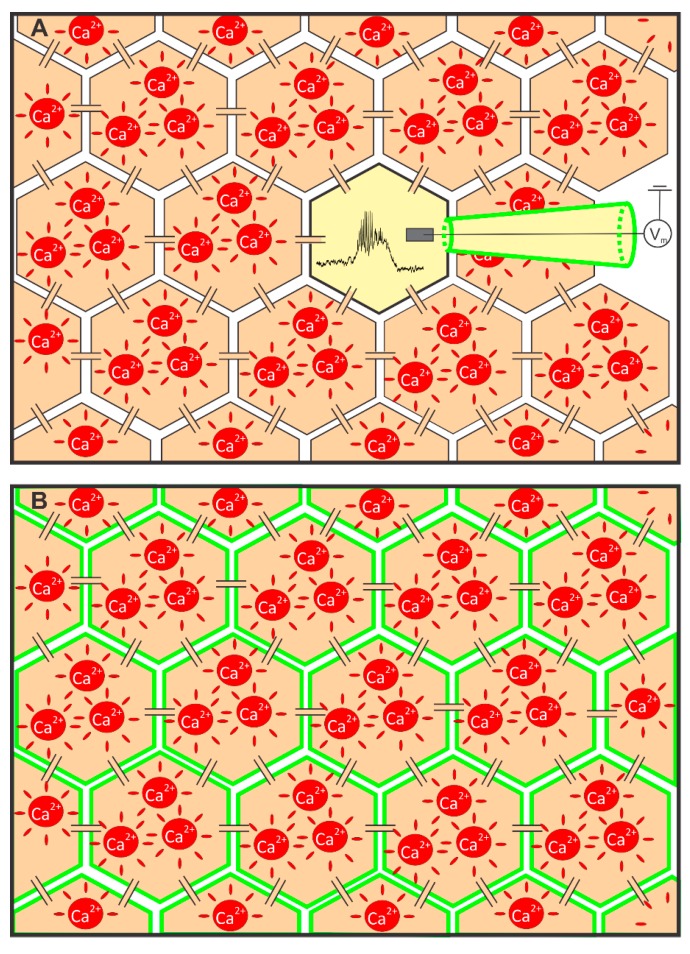
Experimental methods used to simultaneously measure MP and [Ca^2+^]_i_. (**A**) A schematic representation of the whole-cell patch-clamp measurement of MP combined with recording [Ca^2+^]_i_ in the neighboring cells employing a CCD camera. MP from a single cell is monitored via a patch pipette (depicted with green lines), whereas the neighboring cells were loaded with Oregon Green BAPTA-1 AM to monitor changes in [Ca^2+^]_i_; and (**B**) a schematic representation of confocal MP measurement using the voltage sensitive dye Voltage Fluor 2.1 (VF) combined with [Ca^2+^]_i_ recordings using Rhod-2 AM.

In principle, the patch-clamp method electrically isolates a patch of plasmalemma from the external solution [109,110]. This isolation is done by making a tight contact (seal) between a fire-polished glass pipette filled with an intracellular-like electrolyte solution and the surface of the cell. Applying light suction helps to form a tight seal with an electrical resistance as high as 10 GΩ. This seal allows to record current or MP over the patch of membrane while holding MP or current, respectively, at a desired clamped value. Rupturing the patch of membrane isolated by the patch pipette yields the so called whole-cell patch-clamp configuration which connects the cytosol and the pipette interior, thereby allowing clamping of the whole plasmalemma instead of just a small part of it. This approach, which can be applied also to cells within tissue slices, has been effective in measuring MP dynamics in beta cells in mice [68,111] and rats [112], as well as in pituitary cells in mice [113]. It was effectively used to study MP dynamics in spite of inherent equilibration of the cytosol compartment with the pipette content and subsequent wash-out of the cytosol. We have been able to upgrade the classical electrophysiological setup with [Ca^2+^]_i_ imaging using a water-cooled CCD camera and have applied this method on acute mouse pancreas tissue slices (Figure 1). The pancreas tissue slice technique was first introduced by Speier and Rupnik in 2003 [85] as an alternative to most widely-used approaches of isolating islets or single cells [107]. Agarose is injected into the ductal tree and serves as a scaffold allowing to cut pancreas into thin slices. Perhaps the most important functional advantage of this approach is that dyes are not limited mostly to the islet periphery as is the case in isolated islets (e.g., [114]); rather all layers of islets are accessible [87]. This is important since in mice beta cells are located mainly in the islet core [102,103,115,116,117] and therefore relatively inaccessible in isolated islets. Moreover, no enzymes are added and enzymes from the exocrine tissue are inhibited during slicing and mechanical disruption of cells is limited to the outermost layer. In deeper layers, starting from two to three cells below the surface, cells are viable and intact [87]. There are also disadvantages of the tissue slice approach. The islet innervation is discontinued and the perfusion via blood vessels is disrupted and replaced by perifusion which supplies the tissue differently from the *in vivo* situation. To assess the influence of nerves and vessels, an approach closer to the *in vivo* situation is required, such as transplanting islets into the eye [40,118,119,120]. However, the *in vivo* recording inherently lacks the ability of precisely controlling stimulatory conditions. Taken together, at present the tissue slice technique is probably the best compromise or middle ground approach allowing researchers to reliably record [Ca^2+^]_i_ from many beta cells simultaneously.

Generally, two types of calcium indicators are used to monitor [Ca^2+^]_i_, namely genetically-encoded calcium indicators and chemical indicators. Examples of the former are fluorescent proteins derived from GFP or its variants that allow for single wavelength excitation [121], chameleon proteins utilizing FRET-based emission shift upon calcium binding [122]; and auto-luminescent BRET-based luciferase constructs [123]. The chemical indicators, on the other hand, are chemical fluorescent probes which must be loaded into cells in order to report [Ca^2+^]_i_ on site. The most frequently used fluorescent probes are based on BAPTA, the EGTA homologue, which chelates calcium ions and has a high selectivity for calcium. Fluorescent probes are nowadays most widely used in the form of acetoxymethyl (AM) esters. These probes have a masked carboxyl group that chelates calcium ions and can be loaded into cells due to their lipophilic nature. In general, the lipophilicity of different ester probes differs, therefore, in some cases, assistance of amphiphilic polymers (e.g., Pluronic^®^) is required to increase the efficacy of dye loading into cells [124]. When the fluorescent probes are loaded, the intracellular esterases cleave the carboxyl groups form the probes, thereby unmasking the functional part of the dye and therefore rendering the indicator molecule capable to report [Ca^2+^]_i_ changes. Figure 1A depicts the setup when [Ca^2+^]_i_ imaging is combined with the electrophysiological approach in pancreas tissue slices. MP is monitored via a patch-pipette, whereas an AM-based fluorescent probe (in this case Oregon Green 488 BAPTA-1 AM calcium dye (OGB-1, Invitrogen, Eugene, OR, USA)) is loaded into neighboring cells and monitored with a CCD camera [86,87]. This method has many advantages but also some drawbacks. The whole-cell patch-clamp technique has a very high temporal resolution (>1 kHz) and is able to record changes in membrane potential in a range of a few millivolts. On the other hand, only a single cell within an islet can be monitored at a time. [Ca^2+^]_i_ imaging using a CCD camera has, compared to the confocal imaging, a lower spatial resolution. Furthermore, also the temporal resolution is smaller due to unresponsiveness of cells when they are exposed to the fluorescent light for a longer period of time or at higher sampling frequencies [63,87,125,126,127,128,129,130], and the recording times are more limited due to photobleaching triggered by longer exposure times. Finally, a CCD camera-based recording does not allow one to reliably resolve the origin of the signal to a single cell within an islet due to thicker optical sections [86].

**Figure 2:**
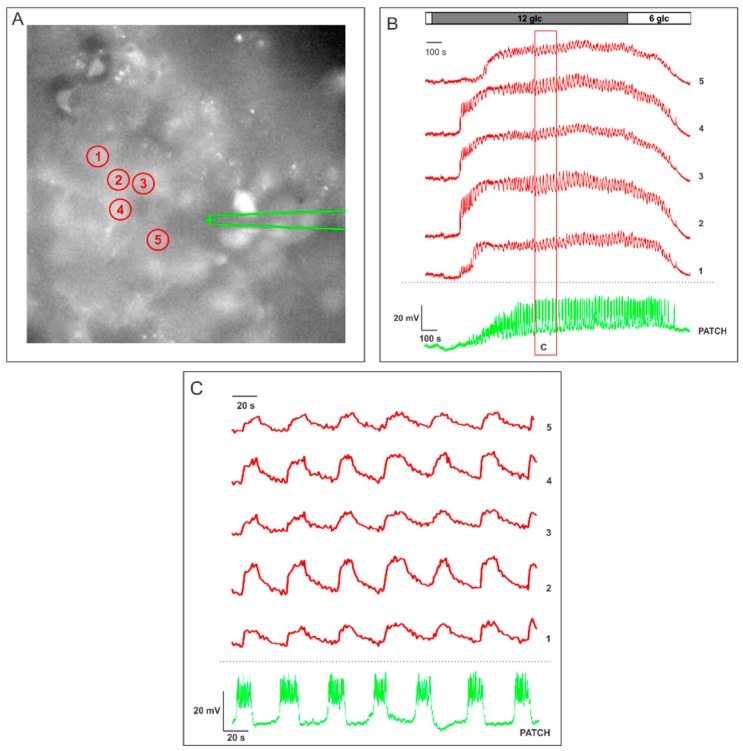
Simultaneous measurement of changes in MP using whole-cell patch-clamp and [Ca^2+^]_i_ using Oregon Green BAPTA-1 AM. (**A**) The [Ca^2+^]_i_ sensitive dye OGB-1 labels intracellular compartments of cells. The numbers indicate cells shown in panels B and C. The patched cell is indicated with +; (**B**) the green trace represents oscillations in MP after increasing the concentration of glucose from 6 to 12 mM. The upper five red traces (1–5) represent [Ca^2+^]_i_ dynamics obtained from the five cells indicated in A. The red rectangle encloses the area shown in panel C under magnification; and (**C**) a more detailed depiction of the response from panel B. Note that each burst in MP (green) is followed by a [Ca^2+^]_i_ (red) oscillation in other cells.

Figure 2 depicts a typical result that can be obtained using a combination of electrophysiology and CCD camera recording in a mouse pancreas tissue slice. When beta cells are exposed to a non-stimulatory concentration of glucose (6 mM), the MP of the patched cell is polarized and the [Ca^2+^]_i_ in surrounding beta cells within the islet is low. After applying a stimulatory glucose concentration (12 mM glucose), beta cells respond in a characteristic pattern that allows for their functional discrimination [131,132,133]. The transient first phase of the response is composed of a depolarization superimposed by frequent individual bursts which can blend into continuous bursting (Figure 2B, trace labelled “patch”). Subsequent to the first phase, a stable second phase consists of intermittent bursts of activity. The neighboring cells respond with a transient increase in [Ca^2+^]_i_ followed by [Ca^2+^]_i_ oscillations superimposed on a sustained plateau (Figure 2B, traces 1–5). Figure 2C shows magnification of the same record revealing that using this approach, the pattern of [Ca^2+^]_i_ in the neighboring cells seems to exactly follow that of the MP [86,87]. MP and [Ca^2+^]_i_ oscillations during the stable second phase have the same frequency and similar shape and are in phase with oscillations in [Ca^2+^]_i_ in mice [127,128]. A careful analysis of Figure 2C reveals that each MP oscillation during the stable second phase is followed by an oscillation in [Ca^2+^]_i_. This delay is well-explained by the fact that MP and [Ca^2+^]_i_ oscillations spread over the islet in a wave-like manner in mice [86,87]. The wave-like nature results in temporal shifts between [Ca^2+^]_i_ oscillations recorded in distant cells (indicated with numbers 1–5 in Figure 2A), with respect to the MP signal in the patched cell (indicated with + in Figure 2A). Unfortunately, this approach lacks the ability to measure MP and [Ca^2+^]_i_ from the same cell, and even more, in many cells simultaneously.

### 2.2. Measuring MP with Novel Voltage-Sensitive Dyes and [Ca^2+^]_i_ with Fluorescent Dyes Using Confocal Microscope

Fluorescence imaging surpasses the limits of the conventional electrophysiological approach and provides a useful tool to map the activity of many cells simultaneously. Due to its advantageously-high sensitivity, [Ca^2+^]_i_ imaging is used most commonly [134] and was successfully employed to indicate the degree of synchronicity between beta cells in mouse [73,127,135,136,137] and human islets [83,103,138,139,140,141,142]. Recently, data on [Ca^2+^]_i_ dynamics in mice provided valuable novel insight into the physiology of beta cells [78,79,80,81,87]. However, since beta cell stimulus-secretion coupling is complex in the sense that it involves many additional steps upstream and downstream from changes in [Ca^2+^]_i_, deducing the whole signaling from the input in the form of glucose and other nutrients to the output in the form of insulin secretion by analyzing changes in [Ca^2+^]_i_ only is not possible. Conceivably, the most important processes upstream of the Ca^2+^ signal involve metabolic pathways generating MCFs and the change in MP which activates VDCCs. In our quest to simultaneously study different parameters, as a first step, we have chosen the combination of MP and [Ca^2+^]_i_ due to its being practically feasible and due to the fact that simultaneous recording of MP and [Ca^2+^]_i_ changes could help further our understanding of the differential effects of various secretagogues that increase [Ca^2+^]_i_ via membrane depolarization and/or via stimulating release from intracellular stores. As mentioned in introduction, recording changes in MP and [Ca^2+^]_i_ together with the most proximal metabolic and distal exocytotic events in tissue slices remains a challenge for the foreseeable future.

Generally, two approaches are used to optically monitor MP in biological membranes. On the one hand, electrochromic dyes modulate the amplitude of the emitted fluorescence due to a shift in their emission spectra provoked by changes in the electric field across the plasma membrane. The main advantage of these dyes are fast response times, but they have a relatively low sensitivity of 10%–28% ΔF/F per 100 mV [143,144]. In the context of beta cell research, this approach was successfully used on cultured cell lines [145,146,147,148]. On the other hand, the fluorescence resonance energy transfer (FRET)-based voltage sensors utilize two components, a lipophilic anion embedded in the membrane and a fluorophore located on one side of the plasma membrane. A change in the transmembrane potential translocates mobile anions between the inner and the outer membrane leaflet. Depending on the MP, translocation of the anions to the side of the membrane on which the immobile fluorophore resides allows for a FRET-based change in fluorescence. Although these FRET-based voltage sensors exhibit a much better sensitivity of up to 80% ΔF/F per 100 mV [149], the translocation of the anion through the lipid bilayer hinders the time resolution of these dyes. Acknowledging the temporal resolution limits, this approach was successfully applied on mouse isolated islets displaying slow [Ca^2+^]_i_ dynamics [150]. An alternative approach that bypasses conventional loading techniques represent the genetically encoded voltage indicators. These are attractive for experimental use since they can be specifically delivered to target tissue and surpass diffusion during conventional loading; however they are limited in their use since they share low sensibility, low brightness, or slow kinetics [151,152,153,154]. Recently, a voltage-sensitive fluorescent protein (VSFP) has been constructed that expresses a shift in its activation curve towards sub-threshold potential changes allowing to reliably optically record local field potentials *in situ* and sensory evoked potentials *in vivo* in mice [155].

Concurrently, a novel family of voltage sensitive dyes was introduced that combines advantages of both electrochromic and FRET-based voltage sensitive dyes. The dye was termed VoltageFluor (VF) and it utilizes a photo-induced electron transfer (PeT) [134,151,156]. Briefly, the fluorophore part of VF localizes to the plasma membrane and its synthetic molecular part protrudes into the lipophilic membrane core and is also called the molecular wire. At the hyperpolarized resting potential, the local electric field promotes electron transfer from the electron donor on the molecular wire to the fluorophore, in turn quenching the fluorescence of the fluorophore. Upon membrane depolarization during activation of a cell, PeT becomes less favorable and, thus, the fluorescence increases due to unquenching. The sensitivity of this system allows for a linear change in fluorescence, with a large sensitivity that can reach up to 48% ΔF/F per 100 mV [151], which is approximately a two-fold improvement over the best dyes of the earlier generations [143].

**Figure 3:**
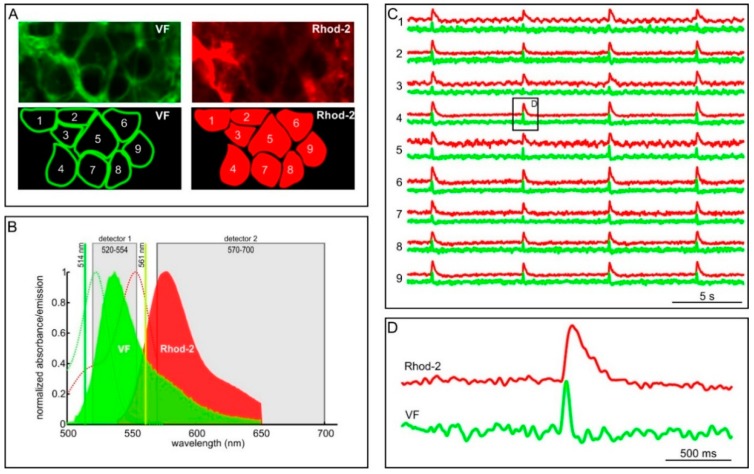
Simultaneously measuring changes in membrane potential using VF and changes in [Ca^2+^]_i_ dynamics using Rhod-2. (**A**) The voltage-sensitive dye VF preferentially labels membranes (upper left), which enables to discriminate single cells (lower left). The [Ca^2+^]_i_ sensitive dye Rhod-2 labels intracellular compartments (upper right). The VF-obtained outlines of cells were used to discriminate Rhod-2 signal of single cells. Numbers are used to indicate cells whose temporal traces are shown in C; (**B**) representation of the experimental setup: two laser lines and two state-of-the-art detectors were used to discriminate signals emitted from VF and signals emitted from Rhod-2; (**C**) [Ca^2+^]_i_ dynamics (red) obtained from 9 cells of a single islet were correlated to simultaneously obtained MP dynamics (green) from the same cells during stimulation with 12 mM glucose and 10 mM tetraethylammonium (TEA). Traces are numbered according to labels in A; and (**D**) a detailed presentation of the response of a cell depicted in C. Note that both signals are noisy and that the [Ca^2+^]_i_ oscillation (red) has different dynamics than the MP oscillation (green).

Profiting from both the superior sensitivity of VF and from the benefits provided by the tissue slice method [85], we were able to scrutinize changes in MP in the mouse islet of Langerhans with unprecedented temporal and spatial resolution (Figure 1B) [86,150]. Moreover, spectral properties of the VF dye enable double-loading of cells with MP and [Ca^2+^]_i_ reporter dyes simultaneously, as depicted in Figure 1B. In this case, VF is restricted to cell membranes, whereas the [Ca^2+^]_i_ reporter dye Rhod-2 is localized in the cell interior. Figure 3B briefly summarizes our experimental setup. A considerable spectral difference between the two dyes, together with the abovementioned spatial separation between the two dyes allowed for a reliable signal discrimination [86]. Recently, a novel PeT-based dye named Berkeley Red Sensor of Transmembrane Potential (BeRST) has been introduced [157]. Compared to the VF, the probe has right-shifted spectral properties and a greatly increased photostability. Due to its spectral properties, BeRST can be used together with green [Ca^2+^]_i_ reporter dyes (e.g., Fluo-4, OGB-1), thus presenting an alternative to the combination of the green MP dyes (e.g., VF) and the red [Ca^2+^]_i_ reporter dyes (e.g., Rhod-2). In contrast with the CCD camera-based recording, confocal microscopy allows for subcellular discrimination of changes in MP and [Ca^2+^]_i_ in many cells within an islet of Langerhans. Cellular activity is seen as oscillatory deflections from baseline for both the MP signal (green, Figure 3C) and the [Ca^2+^]_i_ signal (red, Figure 3C). A detailed analysis revealed that the shape of the MP oscillations differs considerably from that of the [Ca^2+^]_i_ oscillations. Furthermore, in every cell, the increase in MP typically precedes the increase in [Ca^2+^]_i_ by >100 ms [86]. Finally, a multilayer network representation and analysis of the MP and [Ca^2+^]_i_ signal propagation assessed in mice by means of our double stain paradigm showed that the largest delays between the MP and the [Ca^2+^]_i_ signal are present in the most connected cells in the tissue [80]. This observation can be a consequence of various reasons that are principally related with the well-known heterogeneity of beta cells [60,65]. Presuming that the largest cells with the highest number of gap-junctions on their cell surfaces are the most interconnected cells in the functional network, the finding could be a consequence of differences in the abundance of cytosolic calcium buffering proteins. More specifically, a higher number of cytosolic buffers in larger cells would evoke a slower rise in [Ca^2+^]_i_. Alternatively, the slower rise could be a consequence of more active pumping into the ER and out of the cells, which has recently been suggested by the higher energy consumption found in the most connected cells [80]. These properties remained undiscovered in previous studies on cell lines [148] and isolated islets [150].

## 3. Part II: Analytical Methods Used to Analyze Noisy Signals of MP and [Ca^2+^]_i_

When capturing fluorescent time series with a low signal-to-noise ratio (SNR), image denoising is arguably the most important step in pre-processing of data for further analysis. Over the last decade, several methods have been developed to solve the problem for removing noise while preserving the structures and edges of elements in the images [158,159,160]. Current edge-preservation image denoising methods do not perform well on fluorescence time series when analyzing signal oscillations and therefore we have recently developed a new denoising method [161]. State of the art denoising methods, like Non-Local Means (NLM), Block Matching 3D (BM3D), and Locally Adaptive Regression Kernels (LARK), perform noise removal on a single image and do not use temporal information [159]. Additionally, wavelet-based methods can be successfully applied for image denoising [162,163] and have also turned out to be very effective for confocal microscopy [164,165]. However, all parameters in these methods are focused on removal of spatial noise only. By denoising of time series, on the other hand, a lot of information is stored in the temporal domain and this information can be efficiently used for noise removal also in the spatial domain. Therefore, a different set and type of parameters must be available for the fine-tuning of the denoising process in both spatial and temporal domains [166,167]. However, known denoising methods cannot amplify the signal or overlay the signal with a different color.

When dealing with noisy time series data in which we are trying to analyze signal oscillations of MP and [Ca^2+^]_i_, an efficient algorithm is needed which is able to effectively separate noise from the signal. It is important to pre-process data in order to analyze signal characteristics (such as duration and amplitude of an oscillation or time constants for oscillatory upstroke and relaxation), which depend on the frequency of data sampling, spatial resolution, and bleaching of the fluorescent signal. This information is crucial for efficient extraction of signal information. In ideal circumstances, such pre-processing is automated, thereby increasing both efficacy and speed of analysis. However, in practical terms, nowadays the abovementioned parameters are still defined by the user or obtained by fitting in most cases [168,169].

Visualization of signal oscillations of time series data in the spatial domain presents an additional and effective tool for a researcher. To perform such a visualization on noisy time frame videos, advanced denoising is a prerequisite for several reasons. First, oscillations in noisy data can be discriminated only by trained and experienced experts, inherently introducing user bias and unnecessary variability. Moreover, visualization of noisy data does not allow to precisely detect and quantify space-time events. We have solved these issues by our new method, which facilitates the visualization of oscillations by coloring and amplifying the signal to discriminate it from the noisy background.

**Figure 4:**
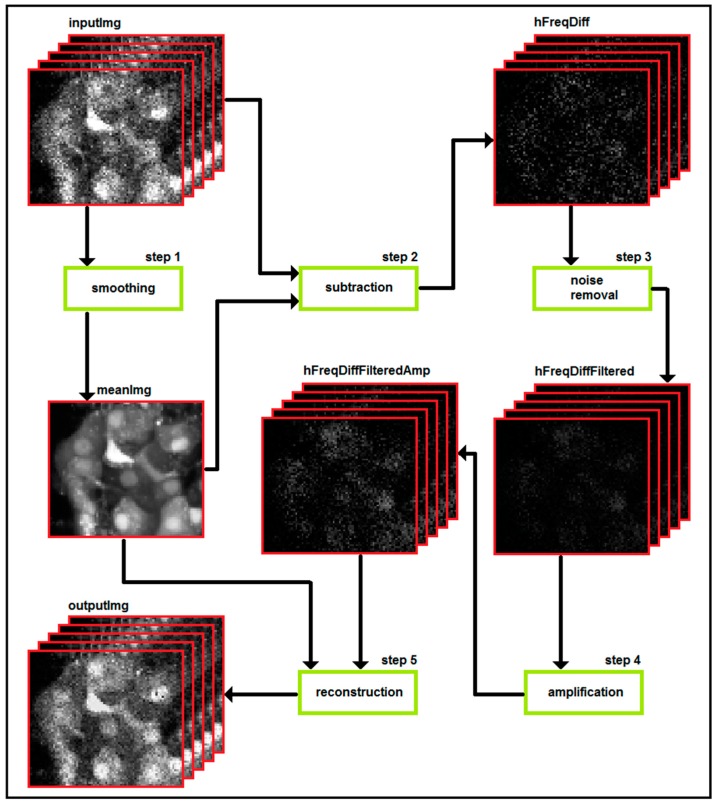
Flowchart representing the algorithm used to pre-process data obtained from confocal imaging of [Ca^2+^]_i_ and MP in pancreas tissue slices. See text for detailed description of the algorithm.

Our method for the improved analysis and monitoring of [Ca^2+^]_i_ and MP oscillations in time-series data (*i.e.*, a video) consists of three steps, as schematically presented in Figure 4. First, detection of changes in fluorescence signal is achieved by decomposing individual images in the time-series (inputImg) into a series of high-frequency differences (hFreqDiff) and a low-frequency mean image (meanImg). This is achieved by smoothing within the temporal domain in order to obtain a representative image of cell structures and, as a consequence, identify potential candidates for noise or signal. Smoothing the images through the time domain into meanImg should, therefore, allow for an efficient estimation of the structural part, whilst the textural part (*i.e.*, inputImg-meanImg) should contain most of the [Ca^2+^]_i_ and MP signal, as well as noise. This is possible since no changes are expected in the positions of the cells over a short period of time. A noise removal filter is then applied on each hFreqDiff, such that the meaningful signal containing information about oscillations is preserved. In the filter, alpha trimming and Gaussian smoothing are used through the temporal and spatial resolution of hFreqDiff and the filtered series without noise is calculated (hFreqDiffFiltered). Since hFreqDiff contains no information about cell structures and edges, all this information is preserved in the final result. Finally, output images are constructed by adding hFreqDiffFiltered to meanImg. This produces a reconstructed image (outputImg) with cell structure and edges stemming from meanImg and the overlaid filtered signal from hFreqDiffFiltered. In the final step, the signal can be amplified by multiplying hFreqDiffFiltered with a selected factor and overlay hFreqDiffFiltered images with different colors to visualize the spatio-temporal dynamics of beta cells more clearly. A more detailed description of this method can be found elsewhere [161].

**Figure 5:**
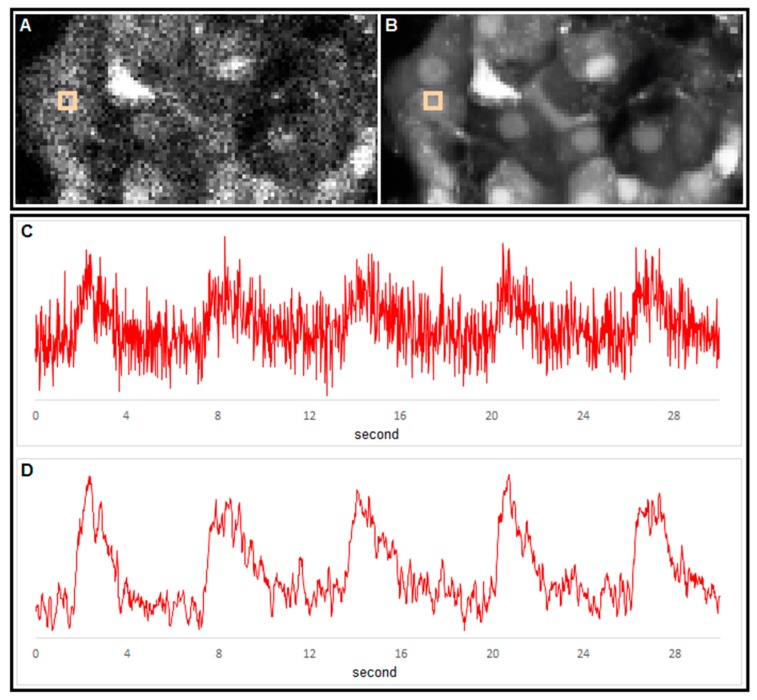
Extraction of spatial and temporal information from [Ca^2+^]_i_ time series using our analytical approach. (**A**) A single image from a [Ca^2+^]_i_ time series. Cells were loaded with OGB-1 AM. The resolution was 256 × 64 pixels @ 50 Hz; (**B**) the average image reveals outlines of cells with cell nuclei stained more intensely than the cytoplasm. The image was averaged over 18,000 frames; (**C**) [Ca^2+^]_i_ signal obtained from 3 × 3 pixels indicated with rectangles in panels A and B; and (**D**) the same signal after denoising. Noise was removed using a Gauss convolution kernel 3 × 3 and a standard deviation of 1 for the spatial domain and moving average filter with a window of length 7 with low cut 1 and high cut 1. The signal has not been amplified.

Figure 5 exhibits a typical high frequency confocal [Ca^2+^]_i_ imaging. The sampling rate during this recording was set at 50 Hz at spatial resolution of 256 × 64 pixels. First, such a high temporal resolution inherently yields noisy data. For example, a single frame (inputImg, Figure 5A) does not provide reliable spatial information about cells. Cell outlines and subcellular structures emerge only after smoothing of images within the temporal domain (meanImg, Figure 5B). Second, in the time domain, raw information extracted from individual pixels appears noisy (Figure 5C), rendering determination of signal properties (e.g., start of upstroke, peak, relaxation) unreliable. Applying the denoising protocol described above significantly improves the signal-to-noise ratio allowing to reliably extract temporal profiles. Since a beta cell is typically covered by approximately 300–400 pixels, the described approach allows to extract information from subcellular structures and to compare [Ca^2+^]_i_ responses from several different cellular compartments.

**Figure 6:**
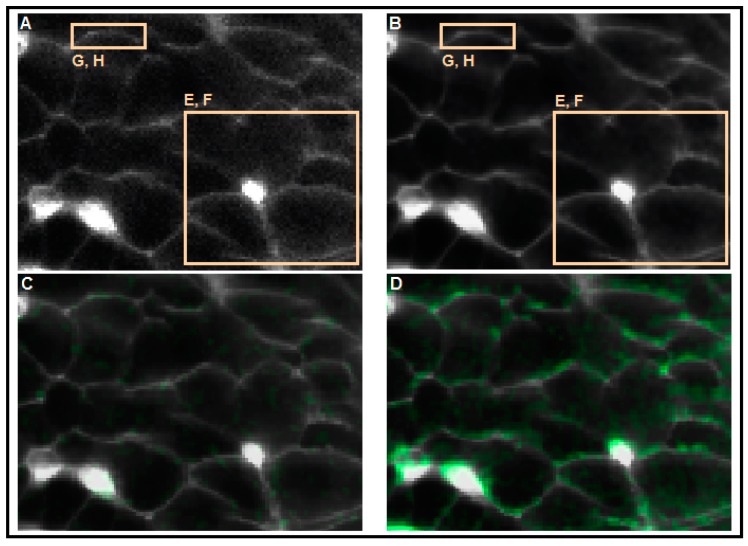

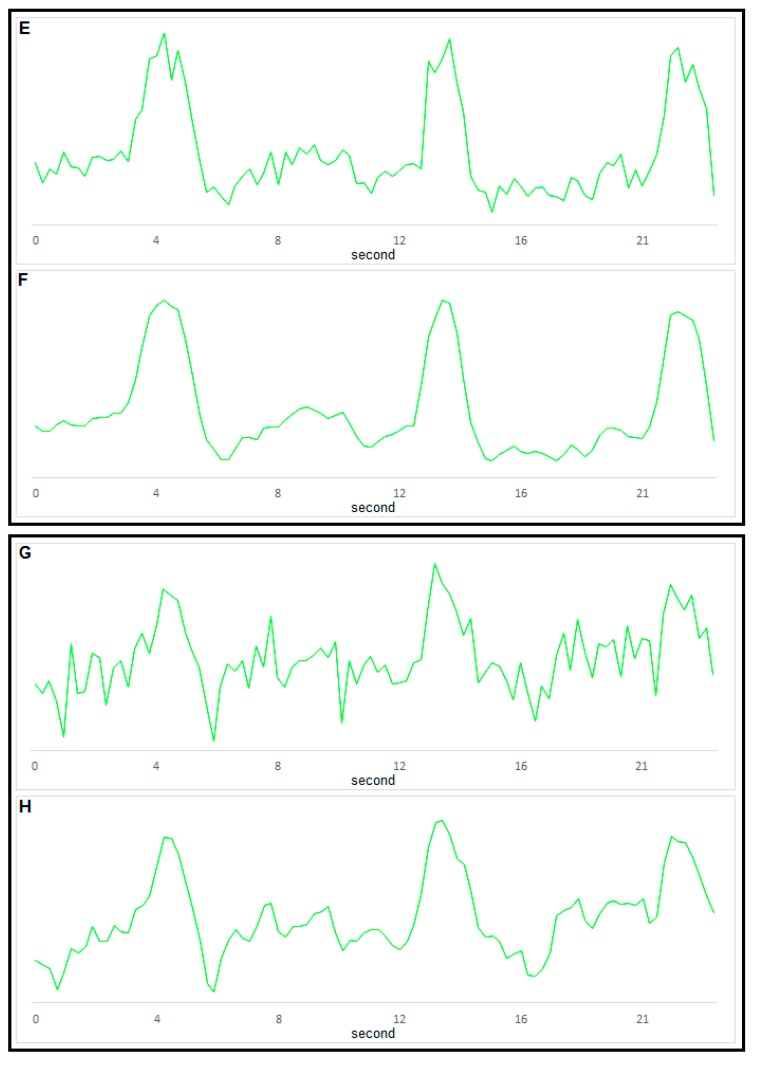
Extraction of spatial and temporal information from MP time series using our analytical approach. (**A**) Single image from a MP time series. Cell membranes were loaded with the membrane potential probe VF. The resolution was 256 × 128 pixels @ 4 fps; (**B**) the average image emphasizes cell outlines. The image was averaged over 720 frames; (**C**) visualization of MP change before onset of MP deflection. hFreqDiffFiltered is shown in green and meanImg in gray; (**D**) visualization of MP change (green) during MP deflection. Colors as on panel C; (**E**) the MP signal from the larger area enclosed by the rectangle in panels A and B before denoising; (**F**) the MP signal from the respective ROI after denoising; (**G**) the MP signal from the smaller area enclosed by the rectangle in panels A and B before denoising; and (**H**) the MP signal from the respective ROI after denoising. The noise was removed using a Gauss convolution kernel 3 × 3 and a standard deviation of 1 for the spatial domain and moving average filter with a window of length two. The signal has been amplified by a factor of four.

Due to the lower sensitivity of the MP probes compared with [Ca^2+^]_i_ indicators and to the smaller excitable area (thin membrane), high frequency confocal imaging of MP yields data that are even more noisy. Figure 6 depicts a typical result from confocal imaging of MP in beta cells during stimulation with 10 mM glucose. The plasmalemma is hardly distinguishable on individual frames, but becomes clearly visible when images are averaged within the temporal domain (Figure 6A,B, respectively). The MP signal from area covering few cells or a small part of plasmalemma (square, Figure 6A,B) is exceedingly noisy in the time domain (Figure 6E,G). Upon denoising, three oscillatory deflections clearly emerge (Figure 6F,H). Comparing this result with the one we obtained with the patch clamp technique (Figure 2), one can notice that bursts lack the superimposed spikes, most probably due to the sampling frequency used during the optical sampling of the MP. Importantly, this denoising protocol allows us to visualize the MP deflections in the spatial domain (Figure 6C,D). Such a visualization is very helpful in analyzing the spreading of the depolarization over many beta cells [86].

## 4. Conclusions

Since its advent, confocal imaging has helped us a lot in elucidating the normal and pathological responses to various stimuli in beta cells as well as in other cells in the islets of Langerhans and many other cell types. Two issues have longed remained unaddressed due to our inability to record simultaneously from many cells at a time and due to our inability to simultaneously capture signals at different steps along the stimulus-secretion response: first, what are the *in situ* properties of the beta cell response at a population level and second, what is the exact relationship between the various signals along the stimulus-secretion response. Recent advances in indicator and detector sensitivity, together with the tissue slice method and the analytical tools to filter and denoise experimentally obtained data and to understand them within the realm of complex network theory have enabled us to start addressing these issues. To be precise, the proposed methodology facilitates the exact extraction of the interaction patterns among beta cells thereby providing a firm description of the functional organization within the islets of Langerhans. Most importantly, by this means not only the physiology of beta cells can be assessed at a higher organizational level, but also fertile ground is provided for drawing a line between normal and pathological function and predict or detect the development of diabetes mellitus. Namely, recent studies put forward the idea of impaired cell-to-cell pathways in both type-1 and type-2 diabetes [71,83,170,171,172], and even suggest that modulations of gap-junctional communication might lead to the development of novel diabetes therapies [71,84]. To make a step further in our understanding of beta cell physiology, in the future the above approaches need to be applied and validated on human tissue. Moreover, some new approaches need to be developed, for instance approaches to track the exocytotic process simultaneously with the more upstream changes in MCFs, MP and [Ca^2+^]_i_.
